# Carbohydrate and lipid metabolism in neonates and children born SGA. A systematic review and metanalysis

**DOI:** 10.1007/s12020-025-04402-9

**Published:** 2025-09-01

**Authors:** Kalliopi Kissoudi, Christos Chatzakis, Panagiotis Christos Mastorakos, Maria Papagianni, Alexandros Sotiriadis, George Mastorakos

**Affiliations:** 1 First Department of Obstetrics and Gynecology, Aristotle University of Thessaloniki, Thessaloniki, 54642 Greece; 2 Second Department of Obstetrics and Gynecology, Aristotle University of Thessaloniki, Thessaloniki, 54642 Greece; 3 Leeds University, Leeds, UK; 4 Department of Nutrition and Dietetics, School of Physical Education, Sport Science and Dietetics, University of Thessaly, 42100 Trikala, Greece; 5 Unit of Endocrinology, Diabetes Mellitus and Metabolism, Aretaieion Hospital, Athens Medical School, National and Kapodistrian University of Athens, Athens, 11528 Greece

**Keywords:** Small for gestational age, Carbohydrate metabolism, Lipid metabolism, Neonates, Children

## Abstract

**Purpose:**

Being born small for gestational age (SGA) is a marker of adverse intrauterine environment and is associated with metabolic disorders in adulthood. The present metanalysis compares the carbohydrate and lipid metabolism between neonates and pre- and post- pubertal children born SGA and their appropriate for gestational age (AGA) -born peers.

**Methods:**

A systematic search was conducted in PubMed, Cochrane, and Scopus databases to identify observational studies comparing carbohydrate and lipidemic profiles in neonates and children born SGA vs. AGA. Data were extracted on insulin, glucose, total cholesterol, HDL, LDL cholesterol and triglyceride concentration. Standardized mean differences (SMD) were calculated using random-effects models. The risk of bias was assessed using Newcastle-Ottawa Scale (NOS). Heterogeneity and publication bias were assessed using the I² statistic and Egger’s test, respectively.

**Results:**

Twenty-eight studies (*N* = 8453) were analyzed. SGA neonates had greater triglycerides (SMD: 0.41, 95% CI: 0.19–0.63) and lower HDL cholesterol (SMD: -0.29, 95% CI: -0.45 to -0.13) concentration than AGA neonates. Prepubertal children born SGA showed significantly greater insulin concentration (SMD: 0.33, 95% CI: 0.10–0.57) than those born AGA. No significant differences were found between AGA and SGA neonates, pre- and post-pubertal children in glucose and LDL cholesterol concentration.

**Conclusion:**

Neonates born SGA show greater circulating triglycerides and lower HDL concentration compared to their AGA peers, while prepubertal children born SGA show greater circulating insulin concentration, potentially predisposing them to insulin resistance. These findings showcase the long-term metabolic consequences of adverse intrauterine conditions, which result to SGA offspring, and emphasize the importance of monitoring SGA neonates and children for potential metabolic disorders during their life.

**Supplementary Information:**

The online version contains supplementary material available at 10.1007/s12020-025-04402-9.

## Introduction

Neonatal birth weight reflects multiple aspects of the intrauterine environment [1]. Being born small for gestational age (SGA) serves as a proxy for adverse fetal circumstances and it is associated with high risk for complications which begin in the neonatal period and may evolve into disease in adulthood [1]. The exact prevalence of SGA depends on the definition employed, but it is estimated to represent 10% of all pregnancies [1]. Risk factors for SGA include all parameters which influence fetal development. The latter is determined by various factors arising from the mother, the fetus and the placental function as well as environmental factors [2].

Being born SGA can significantly impact long-term growth and metabolic health following intrauterine fetal epigenetic adaptations [3]. During the neonatal period, approximately one-third of SGA newborns experience hypoglycemia due to reduced glycogen stores, impaired gluconeogenesis and ketogenesis and low fat stores [1]. Neonates and children born SGA may experience slower growth, often being shorter and lighter, with lower fat mass than their appropriate for gestational age (AGA) peers [1]. Fortunately, most of the SGA neonates experience catch-up growth within the first six months [1]. This catch-up growth occurs through compensatory mechanisms, such as increased appetite and reduced energy expenditure, which may increase later-on the risk of insulin resistance and cardiometabolic diseases [4]. Low birth weight is associated with increased circulating cortisol concentration and resistance to the GH-IGF action [5, 6], an adaptive response to fetal stress, which increases the risk of insulin resistance, hypertension and metabolic syndrome [7]. Additionally, SGA children exhibit abnormal adipose tissue distribution and disrupted function, characterized by altered circulating leptin and other adipokines concentration which regulate appetite and insulin sensitivity [8]. This disruption may contribute to development of insulin resistance, hyperinsulinemia, obesity and associated metabolic disorders [7].

Furthermore, the lipidemic profile of SGA children is characterized by unfavorable lipid concentrations which increase the risk of metabolic disorders. During catch-up growth, SGA children tend to accumulate visceral fat, leading to elevated circulating free fatty acids and alterations in lipoprotein subclasses, including increased and decreased low-density (LDL) and high-density (HDL) lipoprotein cholesterol concentration, respectively [9]. Moreover, the early onset of insulin resistance in these children further exacerbates dyslipidemia by inducing greater triglyceride concentration and lower HDL cholesterol concentration [9].

Children born SGA demonstrate increased risk for metabolic disorders such as insulin resistance, type 2 diabetes and dyslipidemia [10]. Monitoring these profiles allows early detection of pathologic entities, which are crucial for predicting long-term health outcomes, such as metabolic abnormalities in childhood eventually persisting into adulthood, leading to chronic conditions such as cardiovascular disease and obesity [10]. SGA children face challenges that extend beyond low birth weight, with growth and metabolic profiles which predispose them to a range of health risks.

Thus, a metanalysis comparing the circulating concentration of glucose, insulin, triglycerides, total, HDL and LDL cholesterol between neonates and pre- and post- pubertal children born SGA and their AGA peers was undertaken in order to examine whether SGA pathophysiology is associated with an altered metabolic profile at birth as well as later in life.

## Methods

This metanalysis was performed according to the PRISMA statement for metanalyses and is registered with PROSPERO (CRD42023422994).

### Eligibility criteria for studies, participants and outcomes

#### Types of studies

Observational cohort, cross-sectional and case-control studies comparing neonates and prepubertal or pubertal children born SGA to neonates and prepubertal or pubertal children born AGA in terms of metabolic characteristics were considered eligible for inclusion. No country, language or publication date restrictions were imposed.

#### Types of participants

Neonates or/and prepubertal and pubertal children born SGA and neonates or/and prepubertal and pubertal children born AGA, as defined by the authors of primary studies. Only studies pertaining to neonates born at term or late preterm were included. SGA was defined as a birth weight and/or body length either below the 10th percentile, or below the 3rd percentile, or below than 2 standard deviations (SD) from the mean for gestational age.

#### Types of outcome measures

Primary outcomes included: (i) concentration of insulin; (ii) concentration of triglycerides.

Secondary outcomes included: (i) concentration of glucose; (ii) concentration of total cholesterol; (iii) concentration of HDL cholesterol; (iv) concentration of LDL cholesterol.

### Search methods for identification of studies

PubMed, Cochrane Library, and Scopus databases were searched (up to September 30, 2024) for cohort, cross sectional, case control studies comparing neonates or/and prepubertal or pubertal children born SGA to neonates or/and prepubertal or pubertal children born AGA and reporting metabolic characteristics. A combination of the following terms was used: “small for gestational age”, “SGA”, “metabolism”, “metabolic characteristics”, “children”, “neonates”, “outcomes”. Two authors conducted the literature search independently (KK; CC), in case of disagreement, a consensus was reached after discussion. When a consensus could not be reached, a third author offered advice (MP). All studies were compared to avoid duplicating or overlapping samples. In case of the latter, the study with the largest number of events was included. There was no limitation concerning the publication date or the language.

### Study selection

Two authors (KK; CC) assessed the eligibility of all identified citations according to the above mentioned criteria independently. Disagreements between reviewers were resolved by consensus.

### Data collection process and items

Data extraction and assessment of study quality were performed independently by two authors (KK; CC). The study characteristics of each included study were assessed according to a predefined data extraction form. In case of disagreement, a consensus was reached after discussion between the two authors.

### Risk of bias in individual studies

The risk of bias of the included studies was assessed independently by two authors (AS; GM) using the Newcastle-Ottawa Scale (NOS). This scale is developed to assess the quality of cohort, case-control, cross-sectional studies. The studies are judged on eight items, categorized into three groups: selection of study groups; comparability of groups; and ascertainment of either the exposure or outcome of interest. A star is awarded for each quality item; the greatest quality studies are awarded nine stars.

### Summary measures and synthesis of the results

Data were analyzed separately for studies including neonates and those including prepubertal and pubertal children. Three studies (References: 33, 36, and 39) included both neonates and prepubertal children. These studies were counted once in the total number of included studies, while they also contributed data on glucose and insulin concentration to more than one age group. One study (Reference: 36) included both neonates and prepubertal children. This study was counted once in the total number of included studies, while it also contributed data on triglycerides concentration to more than one age group. Data from each study were extracted to note the sample size and the number of events in each study group for nominal outcomes, or the mean and the standard deviation (SD) for continuous outcomes. Data were entered in contingency tables and odds ratio (OR) (95% confidence interval [CI]) or standardized mean differences (SMD) (95% confidence interval [CI]) were estimated for each study as well as a pooled estimate, separately for the groups of neonates and the group of children, weighted by the sample size of each study. Given the non-randomized design and the anticipated heterogeneity of the studies, the summary effect sizes was calculated employing random-effects models. The random-effects model assumes that the true effect size varies between studies and that the included studies represent a random sample of effect sizes that could have been observed. Therefore, this model was chosen because it accounts for comparison of not just variation within studies, but also between studies, thus providing a conservative estimate of the summary statistics with wider confidence intervals (CIs). The heterogeneity between studies was assessed by the estimation of Cochrane’s Q and I^2^ statistic; Egger’s meta-regression test was employed to assess reporting bias in studies when 10 or more studies were available. All analyses were carried out in R (R Foundation for Statistical Computing, Vienna, Austria).

## Results

### Data search results

**Fig. 1:**
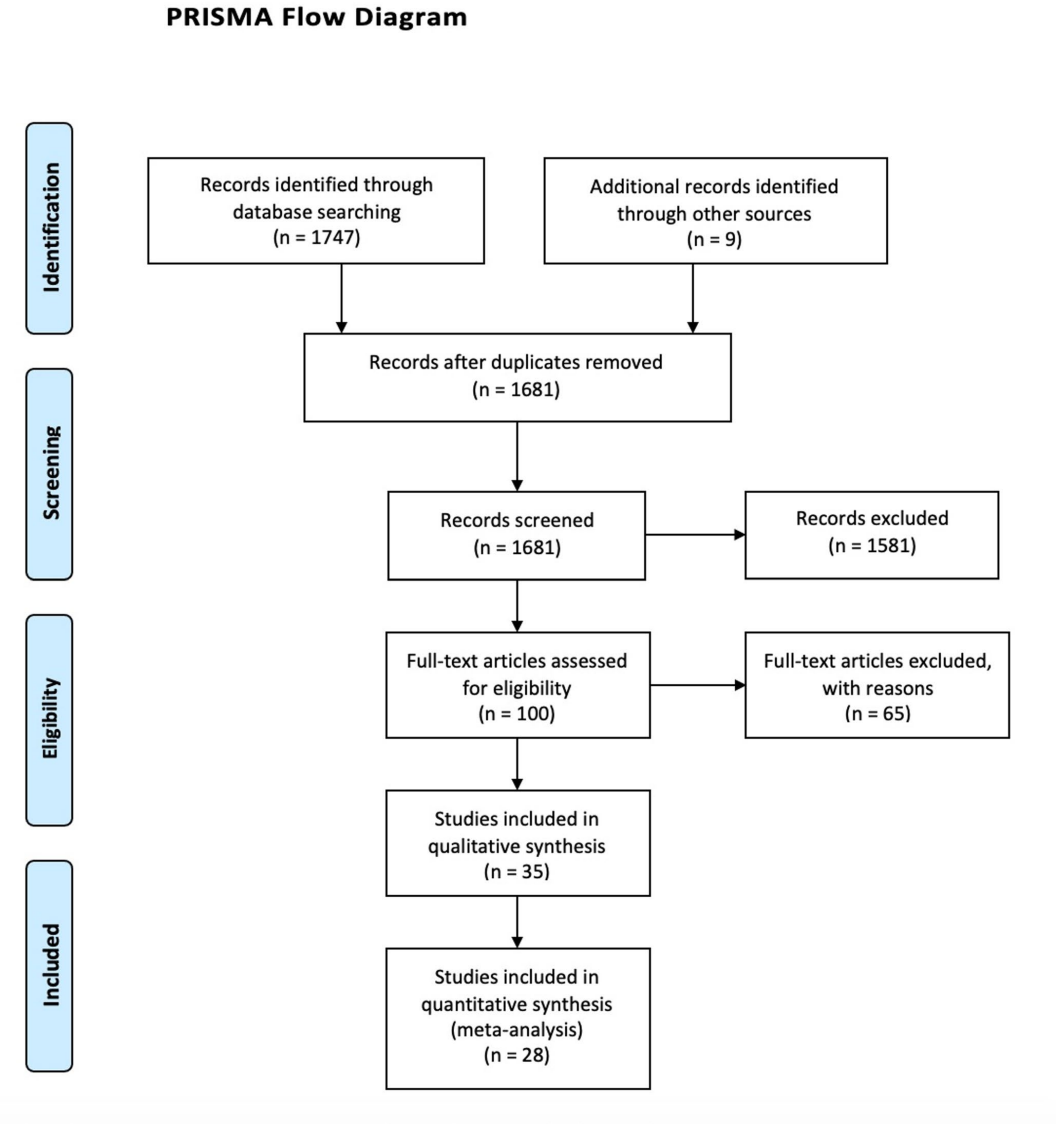
PRISMA Flow diagram of the retrieved studies

The electronic search from the databases yielded 1756 potential studies, of which 1656 were excluded as they were either duplicates, or the title or the abstract indicated that they did not meet the inclusion criteria, leaving 100 studies for full-text review. After full manuscript review, 35 studies [11–45] (*N* = 8453) were finally considered (Fig. 1). The characteristics of the included studies are shown in Table 1. Seven studies were not included in the quantitative analysis as they reported no data for the outcomes of interest, leaving 28 studies in the metanalysis. All included studies were non-randomized trials. The excluded studies, along with the reason for their exclusion are shown in Table S1 in S1 Appendix.

**Table 1:** Presentation of all included studies in the systematic review

	Reference; Year	Study type	Sample size	Indexes	Controls	Confounders	Intervention
1	Wang 2007	Case-control study	296	SGA preterm neonates (*n* = 37) with mean GA 33.9± 0.3 weeks and SGA full-term neonates (*n* = 39) with mean GA 38.6± 0.2 weeks studied at 72 h of age	AGA preterm neonates (*n* = 84) with mean GA 33.4± 0.2 weeks and AGA full-term neonates (*n* = 136) with mean GA 38.8± 0.1 weeks studied at 72 h of age	Gender, gestational age, maternal BMI before pregnancy, weight gain during pregnancy, hypertension during pregnancy, maternal age	N/A
2	Gray 2002	Retrospective cohort study	100	SGA preterm neonates (*n* = 86) studied at birth	AGA neonates (*n* = 14) studied at birth	Gestational age, gender, age, postnatal growth, maternal pregnancy-associated hypertension, type of delivery	Milk tolerance test (MTT) at 19.6± 12.1 d (range 1–65 d) after birth
3	Aly 2015	Prospective cohort study	81	SGA late preterm neonates (*n* = 40) with median GA 34 (34–36) weeks studied on the first day and in the second week of age	AGA neonates (*n* = 41) with median GA 35 (34–36) weeks studied on the first day and in the second week of age	Gestational age, gender, maternal past medical illness, neonatal condition (need for respiratory support etc.), pregnancy induced hypertension, type of delivery	N/A
4	Giapros 2017	Prospective case-control study	95	SGA neonates (*n* = 45) with mean GA 36.1±2.1 weeks studied at 12 months of age	AGA neonates (*n* = 50) with mean GA 36.4± 2.7 weeks studied at 12 months of age	Gestational age, gender, socioeconomic characteristics, risk factors for SGA (maternal smoking, hypertension during pregnancy, low weight gain during pregnancy), cathch-up growth, body weight and length, body fat mass	N/A
5	Wang 2020	Prospective cohort study	1522	SGA neonates (*n* = 105) with median GA 39.4 (38.7–40.3) weeks studied at birth	AGA neonates (*n* = 1320) with median GA 39.3 (38.6–40.1) weeks studied at birth	Maternal characteristics (age, GDM/obesity, pregnancy BMI, spontaneous delivery, primiparity, ethnicity), gestational age, gender, neonatal fat mass, sample storage time	N/A
6	Nieto-Diaz 1996	Case-control study	76	IUGR-SGA neonates (*n* = 31) with mean GA 39.1± 1.4 weeks studied at birth	NBW neonates (*n* = 45) with mean GA 39.6± 1.3 weeks studied at birth	Gestational age, gender, maternal height, maternal weight prepreg, weight gain during pregnancy, smoking habit, vaginal delivery, gestational diabetes, hypertension during pregnancy, urinary infection	N/A
7	Hou 2014	Prospective cohort study	2873	SGA neonates (*n* = 83) with median GA 39 (39–40) weeks studied at birth	AGA neonates (*n* = 2236) with median GA 39 (38–40) weeks studied at birth	Gestational age, maternal age, pre-pregnancy BMI, education, annual income, PIH, gender, mode of delivery, IUGR risk factors	N/A
8	Fister 2019	Case-control study	68	SGA neonates (*n* = 43) with mean GA 37.5±2.8 weeks studied in the first month of age	AGA neonates (*n* = 25) with mean GA 38.6±1.8 weeks studied in the first month of age	Gestational age, gender, maternal BMI, IUGR risk factors, type of delivery	N/A
9	Ghirri 2007	Case-control study	• 90 • 52	• SGA neonates(*n* = 45) with GA ≥ 37 weeks studied at birth • SGA neonates (*n* = 26) with GA ≥ 37 studied at 12 months of age	• AGA neonates (*n* = 45) with GA ≥ 37weeks studied at birth • AGA neonates (*n* = 26) with GA ≥ 37 studied at 12 months of age	Gender, gestational age, type of delivery, Catch-up growth, pubertal stage, BMI	N/A
10	Bauer 2011	Prospective cohort study	32	SGA neonates (*n* = 16) with mean GA 35± 1 weeks studied in the first week of age	AGA neonates (*n* = 16) with mean GA 35± 1 weeks studied in the first week of age	Gestational age, gender, Body fat mass	N/A
11	Bazaes 2003	Prospective cohort study	170	SGA neonates (*n* = 136) with median GA 38 (38–39) weeks studied at 48 h of age	AGA neonates (*n* = 34) with median GA 39 (38–40) weeks studied at 48 h of age	Gestational age, gender, maternal Weight gain during pregnancy, maternal age and height, paternal age and height,	N/A
12	Milovanovic 2014	Prospective cohort study	85	SGA neonates (*n* = 23) with mean GA 38.8± 2 weeks studied at birth, at 1 year and at 4 years of age	AGA neonates (*n* = 62) with mean GA 38.9± 1.7 weeks studied at birth, at 1 year and at 4 years of age	Gestational age, gender, maternal height and weight before pregnancy, parity, ethnicity, maternal risk factors for SGA, type of feeding, body fat mass, BMI	OGTT (glucose administration 1.75 g/kg) at 4 yr
13	Martinez-Cordero 2006	Transverse comparative study	100	SGA neonates (*n* = 50) with median GA 38 (37–40) weeks studied at birth	AGA neonates (*n* = 50) with median GA 39 (37–41) weeks studied at birth	Gestational age, gender, body fat, history of diabetes in a second-degree relative, preeclampsia	N/A
14	Diaz 2020	Prospective cohort study	51	SGA neonates (*n* = 21) with mean GA 38.7± 0.3 weeks studied at birth, at 12 months and at 24 months of age	AGA neonates (*n* = 30) with mean GA 40.2± 0.2 weeks studied at birth, at 12 months and at 24 months of age	Gestational age, gender, BMI, body fat mass, maternal characteristics (age, parity, height, pregestational weight and BMI), type of delivery	N/A
15	Soto 2003	Prospective cohort study	108	SGA neonates (*n* = 85) with median GA 37-41weeks studied at 1 year of age	AGA neonates (*n* = 23) with median GA 37-41weeks studied at 1 year of age	Gender, BMI, gestational age, midparental height	sIVGTT Glucose (25% dextrose solution) dose by continuous infusion over 3 min. Blood samples at -5, 0, 1, 3, 5, and 10 min for insulin and glucose at 1 year of age, after an overnight fast
16	Mericq 2005	Prospective cohort study	68	SGA neonates (*n* = 55) with median GA 37-41weeks studied at birth, at 1 year and at 3 years of age	AGA neonates (*n* = 13) with median GA 37-41weeks studied at birth, at 1 year and at 3 years of age	Gestational age, gender, BMI, body fat mass, Postnatal weight gain	Short IV glucose tolerance test (sIVGTT) annually from 1 year to 3yr
17	Veening 2002	Case-control study	53	SGA neonates (*n* = 29) with mean GA 39.4± 1.4 weeks studied prepubertal at mean age 9.0± 1.1 years	AGA neonates (*n* = 24) with mean GA 39.7± 1.4 weeks studied prepubertal at mean age 9.0± 1.1 years	Gestational age, gender, age, body composition (lean body mass, fat mass), family history of diabetes, hypertension and CVD, BMI, catch-up growth	OGTT (1.75 g/kg glucose, max 75 g) and hyperinsulinemic-euglycemic clamp on two separate days after 12 h of overnight fasting
18	Veening 2004	Case-control study	53	SGA neonates (*n* = 29) with mean GA 39.4± 1.4 weeks studied prepubertal at mean age 9.0± 1.1 years	AGA neonates (*n* = 24) with mean GA 39.7± 1.4 weeks studied prepubertal at mean age 9.0± 1.1 years	Gestational age, gender, A age, body composition (lean body mass, fat mass), family history of diabetes, hypertension and CVD	2-hour hyperinsulinemic euglycemic clamp
19	Lopez-Bermejo 2004	Cross-sectional study	69	SGA neonates (*n* = 32) with mean GA 39.5± 1.3 weeks for lean SGA and mean GA 40.3± 0.5 for overweight SGA studied prepubertal at mean age 5.4± 2.9 years	AGA neonates (*n* = 37) with mean GA 39.1±1.9 weeks for lean AGA and mean GA 38.3±1.4 weeks for overweight AGA studied prepubertal at mean age 5.9± 3.0 years	Gestational age, gender, age, BMI, body fat distribution	N/A
20	Giapros 2012	Retrospective cohort study	155	SGA neonates (*n* = 42) with mean GA 38.2± 1.1 weeks studied prepubertal at age 5.5–7.5 years	AGA neonates (*n* = 63) with mean GA 38.2± 1.3 weeks studied prepubertal at age 5.5–7.5 years	Gestational age, age, gender, BMI, body fat mass, maternal weight before pregnancy, maternal weight gain during pregnancy, hypertensive disease of pregnancy, maternal BMI, maternal age	N/A
21	Evangelidou 2007	Case-control study	70	SGA neonates (*n* = 35) with mean GA 37.0± 1.3 weeks studied prepubertal at age 6–8 years	AGA neonates (*n* = 35) with mean GA 37.9± 1.4 weeks studied prepubertal at age 6–8 years	Age, gender, body weight, body height, BMI, risk factors for IUGR (e.g. hypertensive disease of pregnancy, placental insufficiency, low maternal weight gain during pregnancy)	N/A
22	Stawerska 2016	Case-control study	134	SGA neonates with mean GA 39.6± 0.78 weeks studied prepubertal with normal -stature (*n* = 78) and short-stature (*n* = 14) at mean age 6.9± 1.33 years	AGA neonates with mean GA 39.5± 0.52 weeks studied prepubertal with normal-stature (*n* = 17) and short-stature (*n* = 25) at mean age 7.52± 1.89 years	Gender, gestational age, chronological age, BMI, height, body fat mass	N/A
23	Mohn 2007	Cross-sectional study	48	SGA neonates studied prepubertal with normal-stature (*n* = 19) and short-stature (*n* = 16) at mean age 4.33± 1.88 years	AGA (*n* = 13) neonates studied prepubertal at mean age 3.43± 1.34 years	Gestational age, gender, BMI, fat mass, catch-up growth	N/A
24	Bluskova 2014	Case-control study	98	SGA neonates (*n* = 31) studied prepubertal at age 3-10.9 years	AGA neonates (*n* = 31) studied prepubertal at age 3-10.9 years	Gender, gestational age, age, BMI, IUGR, short stature, catch-up growth status, family history of metabolic disorder	N/A
25	Chiavaroli 2009	Cross-sectional study	57	SGA neonates (*n* = 26) with GA ≥37w studied prepubertal at mean age 6.2± 2 years	AGA neonates (*n* = 15) with GA ≥37w studied prepubertal at mean age 6.3± 2.2 years	Gestational age, gender, age, BMI, fat mass	N/A
26	Starnberg 2019	Prospective longitudinal cohort study	380	Marginally LBW neonates (*n* = 285) with mean GA 38± 1.1 weeks studied prepubertal at age 3.5 years and 7 years	NBW neonates (*n* = 95) with mean GA 40± 1.2 weeks studied prepubertal at age 3.5 years and 7 years	Gestational age, gender, postnatal growth, parental characteristics (maternal and paternal BMI, Maternal ethnicity, age, education), family history of hypertension, diabetes, CVD, IUGR risk factors	N/A
27	Korpysz 2021	Retrospective cohort study	89	SGA neonates (*n* = 30) with mean GA 39.2 weeks studied prepubertal at age 6–7 years	AGA preterm neonates (*n* = 22) with mean GA 33.6 weeks studied at age 6–7 years	Gestational age, gender, age, BMI, Body fat mass, maternal characteristics	N/A
28	Chiavaroli 2014	Cohort study	90	SGA neonates (*n* = 24) with mean GA 39.4±1.4 weeks studied prepubertal at mean age 8.4± 1.4 years	AGA neonates (*n* = 35) with mean GA 39.6± 1.2 weeks studied prepubertal at mean age 8.4± 1.4 years	Gestational age, gender, age, pubertal stage, BMI, height, weight, catch-up growth, body fat mass	N/A
29	Torre 2008	Case-control study	78	CGB-SGA neonates (*n* = 26) and NCGB-SGA neonates (*n* = 26) studied prepubertal at mean age 8.2± 2.8 years and 8.0± 2.8 years respectively	AGA neonates (*n* = 26) studied prepubertal at mean age 7.3± 2.0 years	Gender, age, gestational age, BMI, height, Body fat mass	N/A
30	Cianfarani 2003	Case-control study	135	SGA neonates (*n* = 82) studied prepubertal at mean age 8.6± 3.5 years	AGA neonates (*n* = 53) studied prepubertal at mean age 9.3± 3.3 years	Gestational age, gender, age, Pubertal stage, mid-parental height, BMI	N/A
31	Decsi 1999	Cohort study	32	SGA neonates (*n* = 16) with mean GA 37.9± 1.2 weeks studied pubertal at mean age 9.6± 1.5 years	Preterm neonates (*n* = 16) with GA 34.6± 1.3 weeks studied pubertal at mean age 9.8± 1.4 years	Gestational age, gender, age, BMI, body fat content	N/A
32	Kaneshi 2007	Cohort study	330	Small birth weight neonates (*n* = 33) studied prepubertal and pubertal at age 7–12 years	Middle birth weight neonates (*n* = 264) studied prepubertal and pubertal at age 7–12 years	Gestational age, gender, Age (prepubertal vs. pubertal), postnatal catch-up, BMI	N/A
33	Kistner 2012	Case-control study	77	SGA neonates (*n* = 26) with median GA 40 (37–41) weeks studied prepubertal at median age 9.8 (8.5–10) years	AGA neonates (*n* = 30) with median GA 40 (38–42) weeks studied at 9.8 (9.1–10) years	Gestational age, BMI, age, maternal age, smoking, hypertension/preeclampsia), maternal and paternal anthropometric characteristics, IUGR risk factors, catch-up growth	OGTT 1.75 g/kg max 75 g after a 10–12 h overnight fast
34	Crume 2014	Retrospective cohort study	506	IUGR neonates (*n* = 42) with mean GA 38.6± 1.4 weeks studied pubertal at mean age 10.9± 1.6 years	Unexposed to IUGR neonates (*n* = 464) with mean GA 38.9± 2.1 weeks studied pubertal at mean age 10.6± 1.3 years	Gestational age, gender, age, tanner stage, BMI, ethnicity, activity levels, total energy intake from fat, perinatal exposure factors (e.g., preeclampsia), current socioeconomic status, maternal age at birth, maternal pre-pregnancy BMI, maternal smoking status during pregnancy, socioeconomic and lifestyle factors	N/A
35	Li 2001	Cohort study	139	LBW neonates (*n* = 29) studied prepubertal at median age 7.9 (7.3–8.5) years	NBW neonates (*n* = 110) studied prepubertal at median age 8.1 (7.8–8.4) years	Gestational age, age, pubertal stage, gender, ethnicity, total fat mass	Tolbutamide-modified frequently sampled intravenous glucose tolerance test after an overnight fast

### Assessment of the quality of included studies

The methodological quality of studies was assessed using the Newcastle-Ottawa Scale (NOS). The rating of the included studies according to the NOS is shown in Table 2. Risk of bias was overall low for 4 of the included studies, moderate for 11 studies and high for the other 20. Bias due to confounding and bias in selection of the reported result were the two most prominent reasons of bias in the included studies, as there were no pre-registered protocols to review in most of the studies.

**Table 2:** Risk of bias assessment (NOS) for A: cohort, B: cross-sectional, C: case-control studies

A	Selection				Comparability^e^	Outcome		
Study	Representativeness of the exposed cohort^a^	Selection of the non exposed cohort^b^	Ascertainment of exposure^c^	Incident disease^d^		Assessment of outcome^f^	Length of follow up^g^	Adequacy of Follow up^h^
Gray et al. 2002	C	A	A	A	A	A	A	A
Wang et al. 2020	A	A	A	A	A, B	A	A	A
Hou et al. 2014	A	A	A	A	B	A	A	A
Bauer et al. 2011	D	C	C	A	C	A	A	A
Bazaes et al. 2003	A	A	A	A	B	A	A	A
Milovanovic et al. 2014	C	C	A	A	B	A	A	A
Korpysz et al. 2021	D	C	D	A	C	A	A	D
Giapros et al. 2012	A	A	A	A	A, B	A	A	A
Diaz et al. 2020	A	A	A	A	A, B	A	A	B
Decsi et al. 1999	C	A	A	A	C	A	A	A
Soto et al. 2003	A	A	A	A	C	A	A	A
Mericq et al. 2005	A	A	A	A	C	A	A	A
Crume et al. 2014	A	A	A	A	B	A	A	A
Starnberg et al. 2019	C	B	A	A	A, B	A	A	B
Kaneshi et al. 2007	B	A	C	A	B	A	A	A
Chiavaroli et al.2014	A	A	A	A	B	A	A	B
Li et al. 2001	C	A	C	A	B	A	A	C
Aly et al. 2015	C	A	A	A	A, B	A	A	B

B	Selection				Comparability^m^	Outcome	
Study	Representativenessof the sample^i^	Sample size^j^	Non-respondents^k^	Ascertainment of the exposure^l^		Assessmentof outcome^n^	Statistical test^o^
Lopez-Bermejo et al. 2004	C	A	C	A	B	A	A
Mohn et al. 2007	B	C	C	A	B	A	B
Chiavaroli et al. 2009	C	C	C	A	B	A	B
Martinez-Cordero et al. 2006	C	C	C	A	B	A	B

C	Selection				Comparability^t^	Exposure		
Study	Adequate definition^p^	Representativeness of cases^q^	Selection of controls^r^	Definition of controls^s^		Ascertainment of exposure^u^	Same method of ascertainment for cases and controls^v^	Non-Response rate^w^
Wang et al. 2007	A	A	A	A	A	A	A	A
Veening et al. 2004	A	A	A	A	A, B	A	A	A
Nieto-Diaz et al. 1996	A	A	A	A	B	A	A	A
Fister et al. 2019	A	A	A	A	-	A	A	A
Ghirri et al. 2007	A	A	A	A	A, B	A	A	A
Giapros et al. 2016	A	A	A	A	A, B	A	A	A
Veening et al. 2002	A	A	A	A	B	A	A	A
Cianfarani et al. 2003	A	A	A	A	B	A	A	A
Evangelidou et al. 2007	A	A	A	A	B	A	A	A
Kistner et al. 2012	A	A	A	A	A	A	A	A
Stawerska et al. 2016	A	A	A	A	-	A	A	A
Bluskova et al. 2014	A	A	A	A	B	A	B	A
Torre et al. 2008	A	A	A	A	B	A	A	A

^a^A, truly representative of the average neonates/infants/children/adults; B, somewhat representative of neonates/infants/children/adults; C, selected group; D, no description of the derivation of the cohort

^b^A, drawn from the same source as the exposed cohort (concurrent controls); B, drawn from a different source (historical controls); C, no description of the derivation of the nonexposed cohort

^c^A, secure record (e.g., hospital records); B, structured interview; C, written self-report; D, no description

^d^Demonstration that outcome of interest was not present at the start of the study: A, yes; B, no

^e^Comparability of cohorts on the basis of the design or analysis: A, study controls for gestational age and gender; B, study controls for any additional factor; C, not carried out or not reported

^f^A, independent blind assessment; B, record linkage; C, self-report; D, no description

^g^Was follow-up long enough for outcomes to occur? A, yes; B, no

^h^A, complete follow-up; all subjects were accounted for. B, Subjects lost to follow-up were unlikely to introduce bias because small numbers were lost; >=80% had follow-up, or description was provided of those lost. C, follow-up rate < 80%, and there was no description of those lost. D, no statement

^i^A, truly representative of the average; B, somewhat representative of the average; C, selected group; D, no description of the sampling strategy.

^j^A, justified and satisfactory; B, not justified; C, no information provided

^k^ A, Comparability between respondents and non-respondents characteristics is established, and the response rate is satisfactory; B, The response rate is unsatisfactory, or the comparability between respondents and non-respondents is unsatisfactory; C, no description

^l^ A, Validated measurement tool (clinical record); B, Non-validated measurement tool, but the tool is available or described; C, no description of the measurement tool

^m^ Comparability of subjects on the basis of the design or analysis: A, study controls for gestational age and gender; B, study controls for any additional factor; C, not carried out or not reported

^n^ A, independent blind assessment; B, record linkage; C, self-report; D, no description

^o^ A, the statistical test used to analyze the data is clearly described and appropriate, and the measurement of the association is presented, including confidence intervals and the probability level (p value); B, The statistical test is not appropriate, not described or incomplete

^p^A: yes, with independent validation; B: yes based on records; C: no description

^q^A: obviously representative of the cases; B: potential for selection biases or not stated

^r^A: community controls; B: hospital controls; C, no description

^s^A: no history of disease; B: no description of source

^t^Comparability of cases and controls on the basis of the design or analysis: A: study controls for gestational age and gender; B: study controls for any additional factor

^u^A: secure record; B: structured interview where blind to case/control status; C: interview not blinded to case/control status; D: written self-report; E: no description

^v^A: yes; B: no

^w^A: same rate for both groups; B: non respondents described; C: rate different and no designation

### Parameters of the carbohydrate profile

#### Glycemia (Fig. 2)

**Fig. 2:**
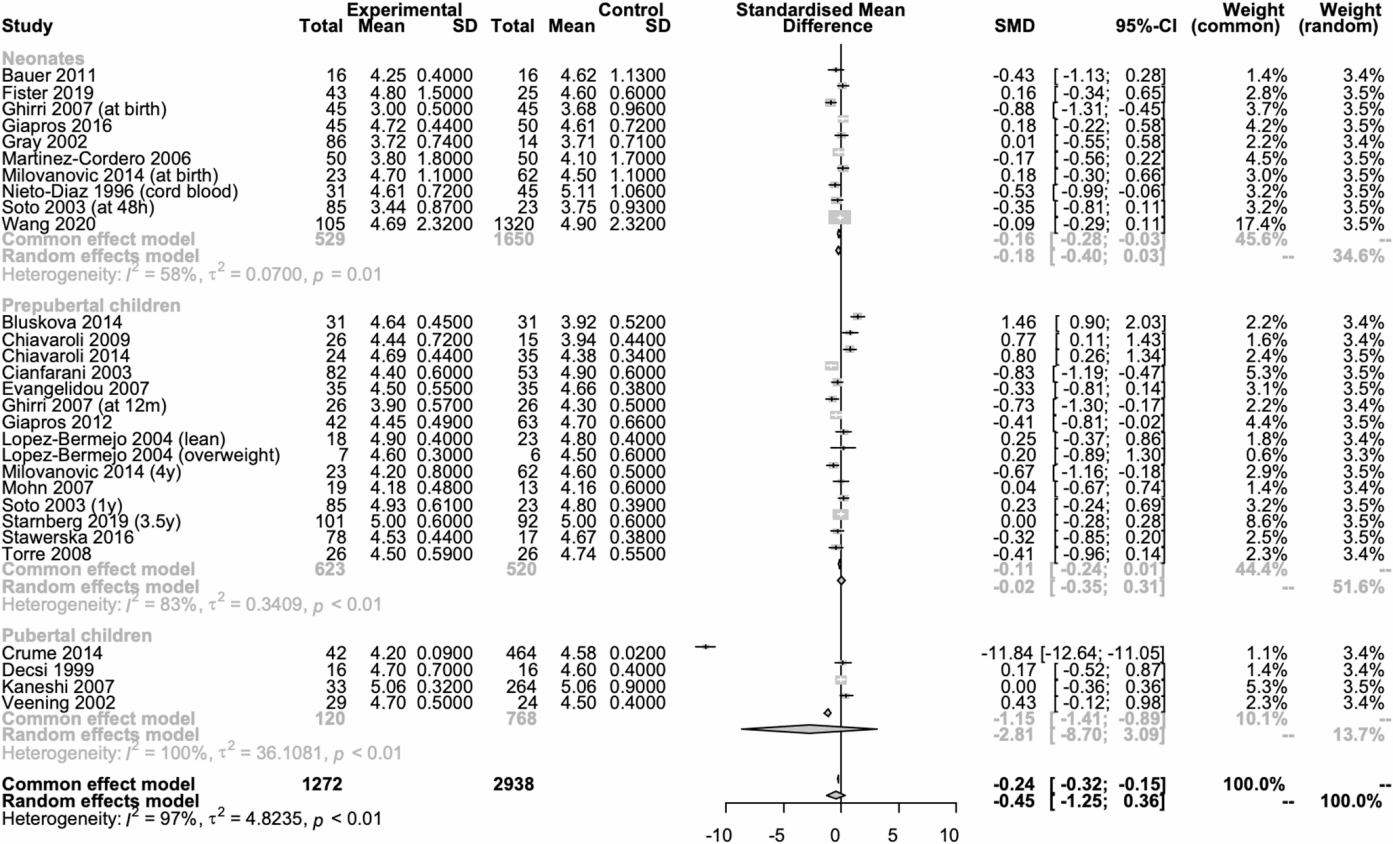
Metanalysis comparing glucose concentration in SGA neonates, prepubertal and pubertal children vs. their AGA peers

Twenty-five studies (*n* = 4210) reported data on glucose concentration [12, 14, 16, 17, 20–22, 24–31, 33, 35, 36, 38–40, 42–45], of which 10 studies (*n* = 2179) were in neonates [33, 35, 36, 38–40, 42–45], 14 (*n* = 1143) in prepubertal children [20–22, 24–31, 33, 36, 39] and 4 (*n* = 888) in pubertal children [12, 14, 16, 17]. In neonates, prepubertal and pubertal children there was no statistically significant difference between those born SGA and those born AGA, SMD: -0,18 (95% CI; -0.4 to 0.03, I2 = 58%); SMD: -0.02 (95% CI; -0.35 to 0.31, I2 = 83%) and SMD: -2.81 (95% CI; -8.70 to 3.09, I2 = 100%) respectively.

#### Insulinemia (Fig. 3)

**Fig. 3:**
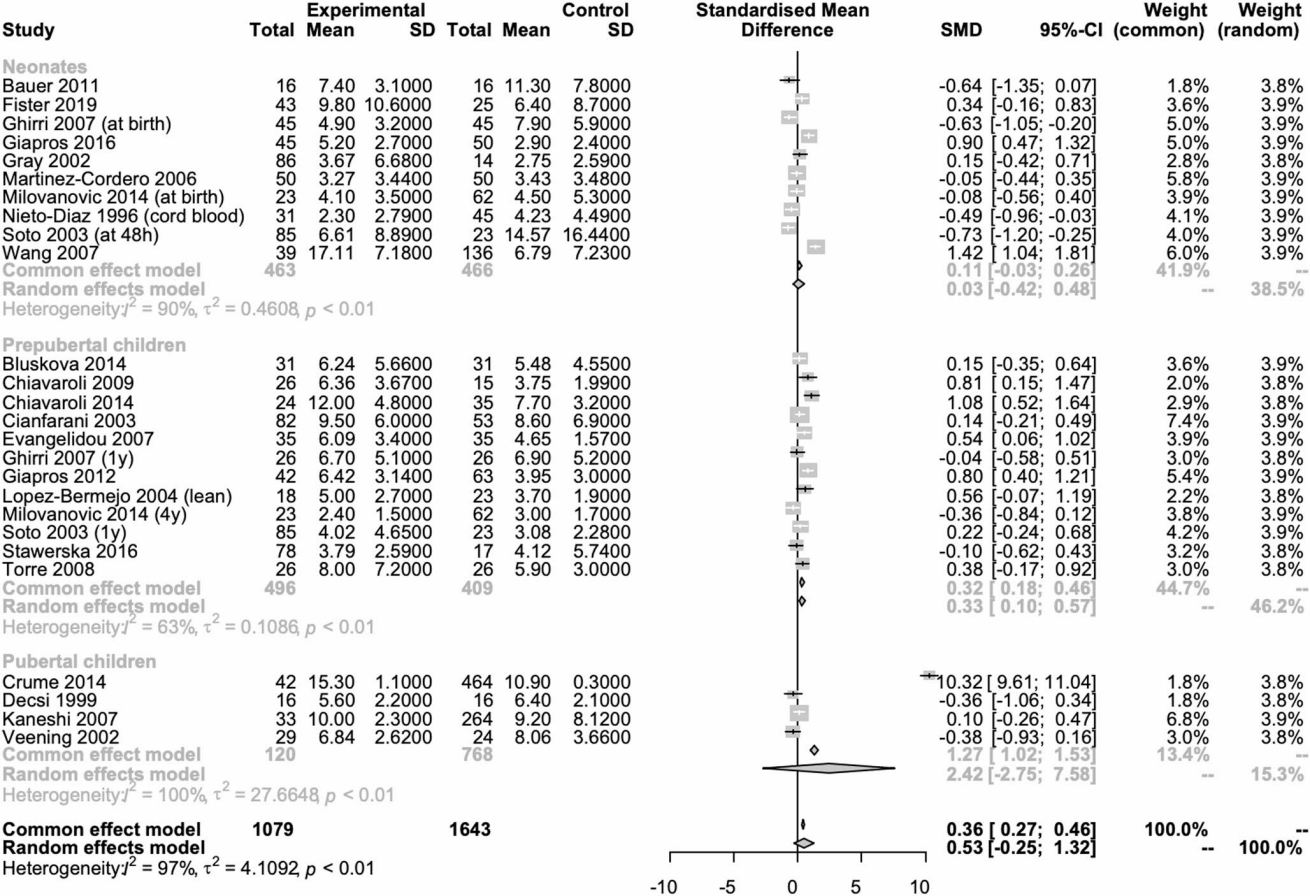
Metanalysis comparing insulin concentration in SGA neonates, prepubertal and pubertal children vs. their AGA peers

Twenty-three studies (*n* = 2722) reported data on insulin concentration [12, 14,16,17, 19–22, 25, 26, 28–31, 33, 35, 36, 38–40, 42, 44, 45], of which 10 studies (*N* = 929) were in neonates [19, 33, 35, 36, 38–40, 42, 44, 45], 12 (*n* = 905) in prepubertal children [20–22, 25, 26, 28–31, 33, 36, 39] and 4 (*n* = 888) in pubertal children [12, 14, 16, 17]. In neonates and pubertal children there was no statistically significant difference between those born SGA and those born AGA, SMD: 0.03 (95% CI; -0.42 to 0.48, I2 = 90%) and SMD: 2.42 (95% CI; -2.75 to 7.58, I2 = 100%) respectively. In prepubertal children, there was a statistically significant difference between those born SGA and those born AGA, SMD: 0.33 (95% CI; 0.10 to 0.57, I2 = 63%).

### Parameters of the lipidemic profile

#### Total cholesterol (Fig. 4)

**Fig. 4:**
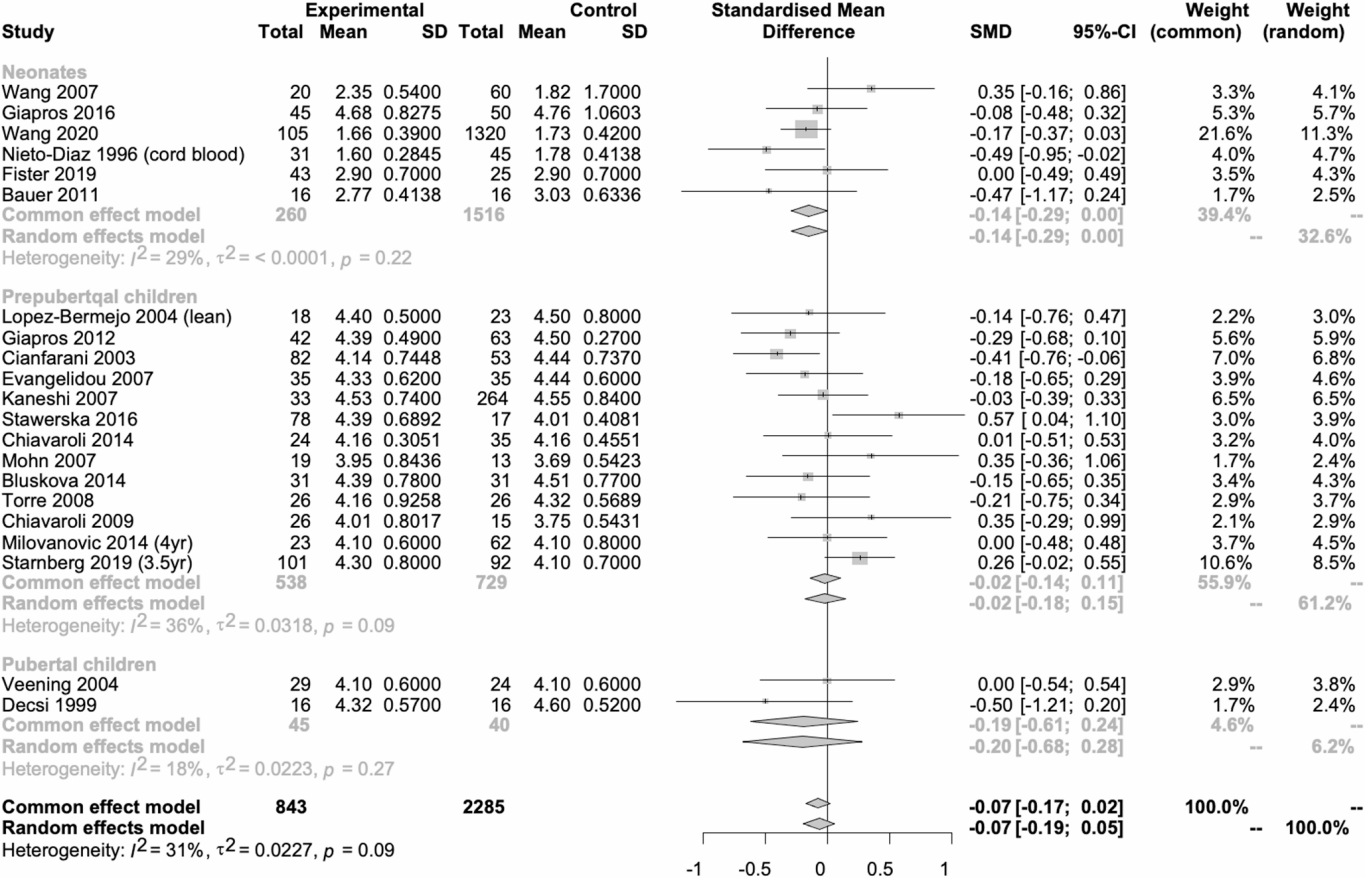
Metanalysis comparing total cholesterol concentration in SGA neonates, prepubertal and pubertal children vs. their AGA peers

Twenty-one studies (*N* = 3128) reported data on the concentration of total cholesterol [16, 17, 19–22, 24–32, 36, 38, 40, 42–44], of which 6 studies (*n* = 1776) were in neonates [19, 38, 40, 42–44], 13 (*n* = 1267) in prepubertal children [16, 20–22, 24–31, 36] and 2 (*n* = 85) in pubertal children [17, 32]. In neonates, prepubertal and pubertal children there was no statistically significant difference between those born SGA and those born AGA, SMD: 0.14 (95% CI; -0.29 to 0.00, I2 = 29%), SMD: -0.02 (95% CI; -0.18 to 0.15, I2 = 36%) and SMD: -0.20 (95% CI; -0.68 to 0.28, I2 = 18%) respectively.

#### High-density lipoproteins (Fig. 5)

**Fig. 5:**
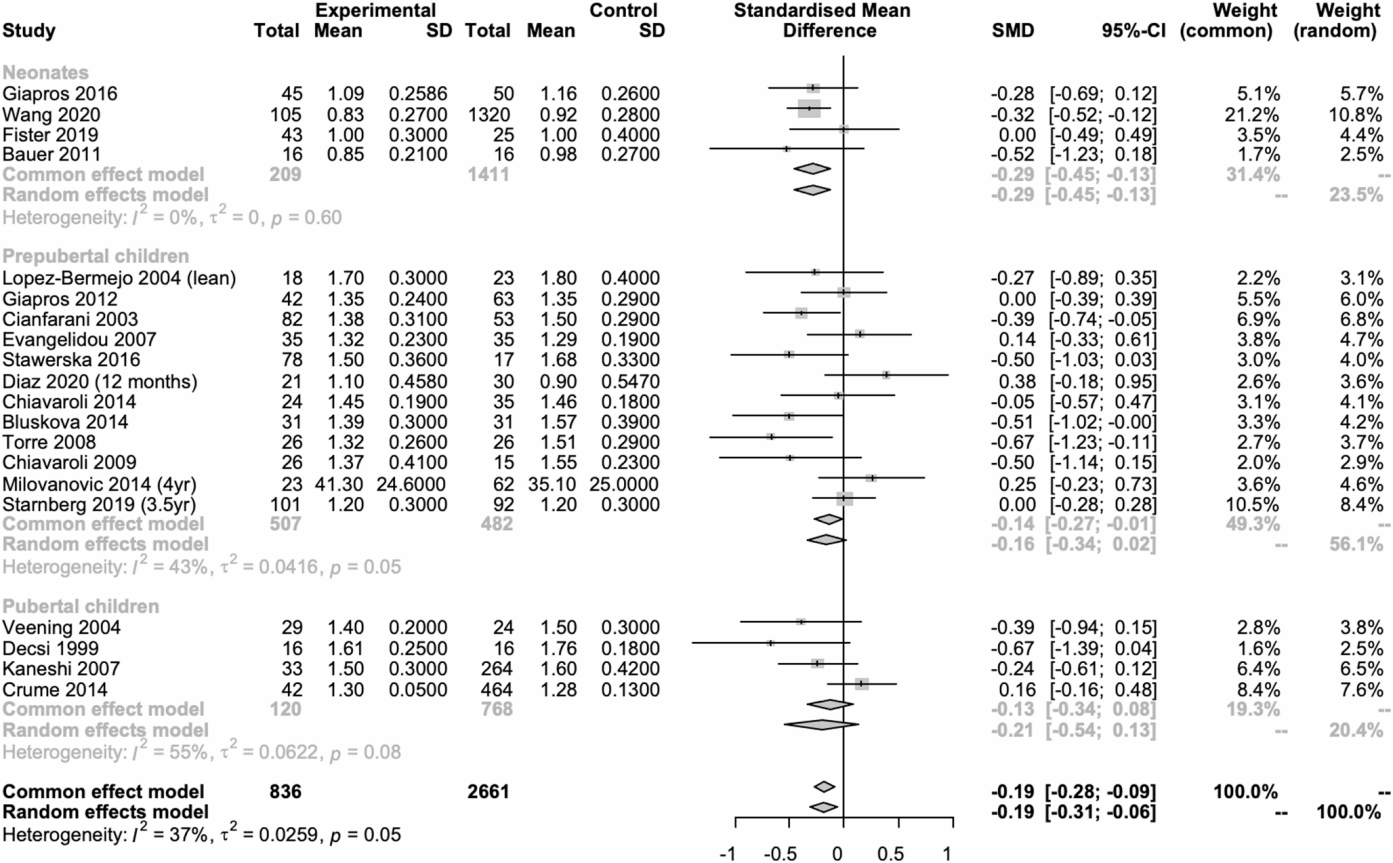
Metanalysis comparing HDL concentration in SGA neonates, prepubertal and pubertal children vs. their AGA peers

Twenty studies (*n* = 3497) reported data on the concentration of HDL [14, 16, 17, 20–22, 24–26, 28–32, 34, 36, 38, 40, 43, 44], of which 4 studies (*n* = 1620) were in neonates [38, 40, 43, 44], and 12 (*n* = 989) in prepubertal children [20–22, 24–26, 28–31, 34, 36] and 4 (*n* = 888) in pubertal children [14, 16, 17, 32]. In neonates there was a statistically significant difference between those born SGA and those born AGA, SMD: -0.29 (95% CI; -0.45 to -0.13, I2 = 0%). In prepubertal and pubertal children there was no statistically significant difference between those born SGA and those born AGA, SMD: -0.16 (95% CI; -0.34 to 0.02, I2 = 43%) and SMD: -0.21 (95% CI; -0.54 to 0.13, I2 = 55%) respectively).

#### Low-density lipoproteins (Fig. 6)

**Fig. 6:**
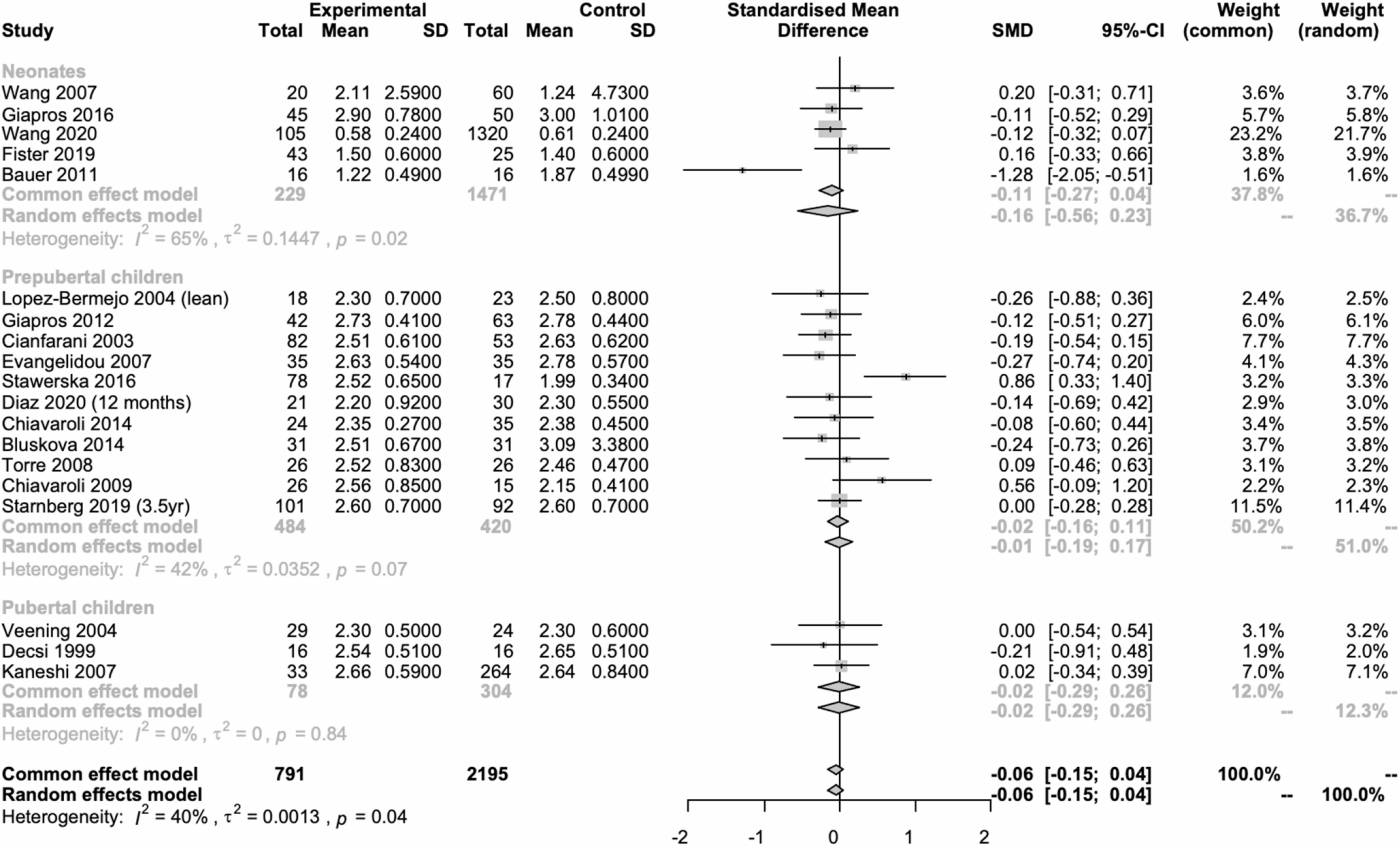
Metanalysis comparing LDL concentration in SGA neonates, prepubertal and pubertal children vs. their AGA peers

Nineteen studies (*n* = 2986) reported data on LDL concentration [16, 17, 19–22, 24–26, 28–32, 34, 38, 40, 43, 44], of which 5 (*n* = 1700) were in neonates [19, 38, 40, 43, 44], and 11 (*n* = 904) in prepubertal children [20–22, 24–26, 28–31, 34] and 3 (*n* = 382) in pubertal children [16, 17, 32]. In neonates, prepubertal and pubertal children there was no statistically significant difference between those born SGA and those born AGA, SMD: -0,16 (95% CI; -0.56 to 0.23, I2 = 65%), SMD: -0.01 (95% CI; -0.19 to 0.17, I2 = 42%).and SMD: -0.02 (95% CI; -0.29 to 0.26, I2 = 0%) respectively.

#### Triglyceridemia (Fig. 7)

**Fig. 7:**
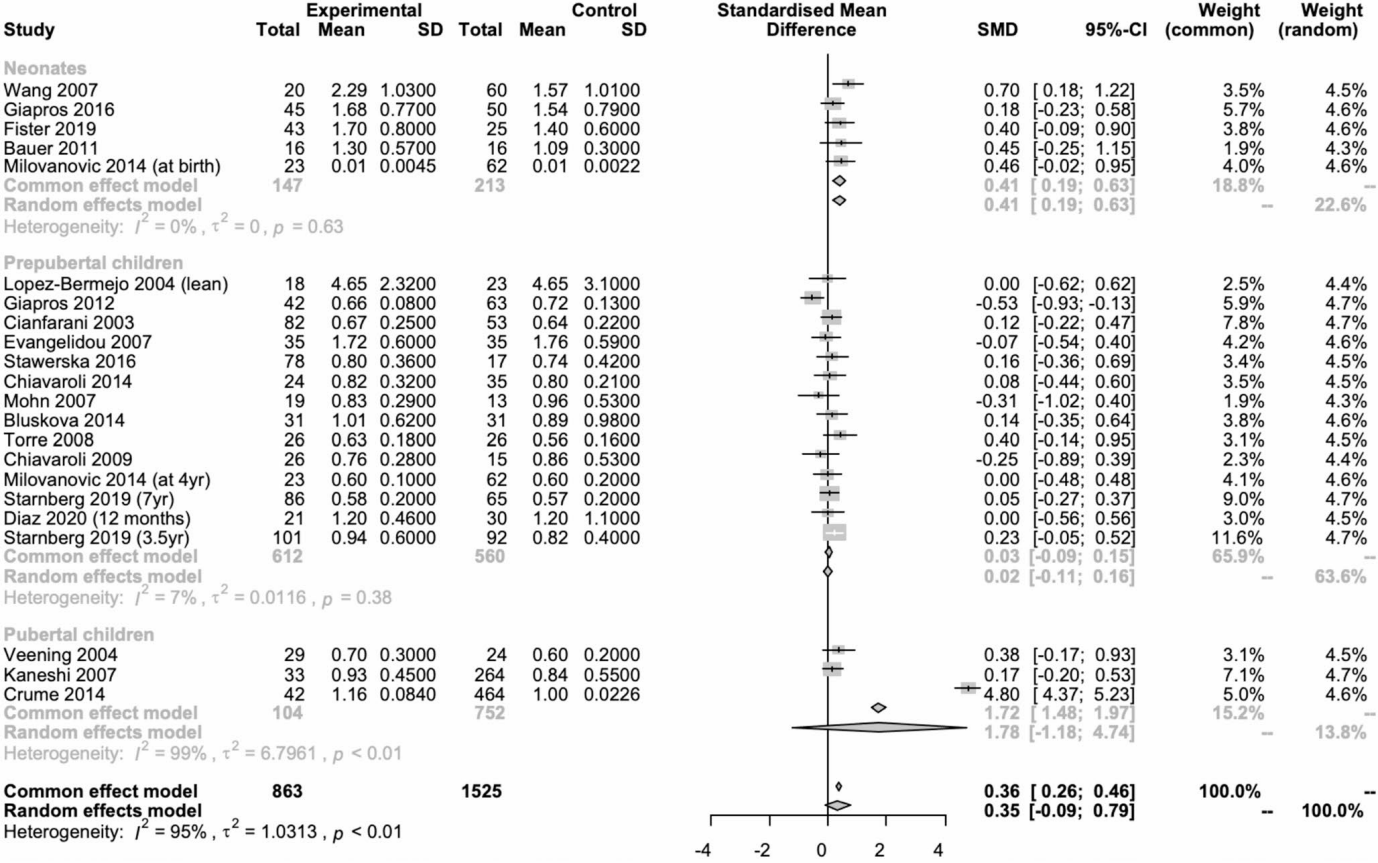
Metanalysis comparing triglyceride concentration in SGA neonates, prepubertal and pubertal children vs. their AGA peers

Twenty studies (*n* = 2388) reported data on triglyceride concentration [14, 16, 19–22, 24–32, 34, 36, 38, 40, 44], of which 5 (*n* = 360) were in neonates [19, 36, 38, 40, 44], 13 (*n* = 1172) in prepubertal children [20–22, 24–31, 34, 36] and 3 (*n* = 856) in pubertal children [14, 16, 32]. In neonates there was a statistically significant difference between those born SGA and those born AGA, SMD: 0.41 (95% CI; 0.19 to 0.63, I2 = 0%). In prepubertal and pubertal children there was no statistically significant difference between those born SGA and those born AGA, SMD: 0.02 (95% CI; -0.11 to 0.16, I2 = 7%) and SMD: 1.78 (95% CI; -1.18 to 4.74, I2 = 99%) respectively.

### Additional analyses

Meta-regression analysis was carried out for all outcomes in the groups of children, assessing the effect of gestational age at birth, BMI and age of children at the time of study. No significant correlations were found between the predictive variables and the included outcomes.

Meta-regression analysis in the group of neonates, assessing the effect of gestational age at birth, was possible only for the outcomes of insulin and glucose concentration, as for the rest of the outcomes less than 10 studies were included. No statistically significant correlation was found between the predictive variable and the included outcomes.

## Discussion

In the present metanalysis, we found that neonates born SGA had greater circulating triglycerides concentration and lower HDL concentration in peripheral blood compared to those born AGA. Furthermore, in prepubertal period, children born SGA showed elevated circulating insulin concentration in peripheral blood, compared to their AGA peers.

In this metanalysis we also found that among neonates, prepubertal and pubertal children born either SGA or AGA, respectively, only prepubertal children born SGA demonstrated greater insulin concentration compared to those born AGA. Several studies confirmed hyperinsulinemia among prepubertal children born SGA [46, 47]. Furthermore, studies showed increased insulin resistance in prepubertal children born SGA, independently of their BMI at the time of the study [46]. The increased insulin concentration observed in children born SGA may reflect either decreased insulin sensitivity or increased insulin resistance and it may progress to metabolic syndrome later in life [7].

The concept of “fetal origin of disease” resulting from fetal and early life events was initially proposed by Barker et al. based on epidemiological observations [3]. In humans, the association between fetal undernutrition and long-term abnormalities in glucose regulation later in life has been demonstrated through follow-up studies of individuals born during the Dutch famine in World War II [48]. Offspring of pregnant women exposed to famine during the late gestational period showed the greatest 2-hour plasma glucose concentration [49]. Phipps et al. confirmed the association between reduced birthweight and glucose intolerance in adult life [50]. The exact mechanisms behind altered insulin tolerance in individuals born SGA are not clear. The ‘thrifty phenotype hypothesis’ has been proposed as an explanation mechanism for the association between low birth weight and metabolic dysregulation, which may progress to type 2 diabetes later in life [51]. It is considered as an adaptive mechanism of the fetus found in a hostile environment. This mechanism is theorized to promote survival, yet it predisposes the fetus to chronic disease later in life, due to permanent epigenetic changes in glucose-insulin metabolism [10]. A redistribution of oxygen and nutrients, especially glucose, is activated to protect the brain, heart and adrenals. As a result, several organs such as the pancreas display irreversible histological and functional alterations, and the organism is conditioned to the development of insulin resistance [7, 51].

In the present metanalysis, no differences in glucose concentration between neonates, prepubertal and pubertal SGA children compared to AGA peers were observed. Regarding neonates born SGA, hypoglycemia is common, due to their reduced hepatic glycogen and fat stores, along with inefficient gluconeogenesis and ketogenesis [1]. Studies in prepubertal children born SGA have shown that, despite being more insulin resistant [46, 47], their blood glucose concentration is not statistically different compared to their AGA peers, either in fasting conditions [52] or during an oral glucose tolerance test (OGTT) [12]. Children born SGA show reduced insulin sensitivity along with compensatory hyperinsulinemia to maintain normoglycemia [46]. A metanalysis [53] comparing prepubertal and pubertal children born SGA to non-SGA subjects, showed no statistically significant difference in fasting glucose and OGTT-2 h glucose concentration between the two groups in the prepubertal group, although there was an upward tendency in OGTT-2 h glucose concentration in prepubertal SGA children. In the pubertal group, this concentration was significantly greater in SGA children. Eventually, accentuation of the glucose metabolism impairment in children born SGA may develop during prepubertal age.

In the present metanalysis, no statistically significant difference in total cholesterol concentration was observed between neonates, prepubertal and pubertal SGA children and their respective AGA peers. Study findings from observational studies regarding total cholesterol concentration in the SGA group of children have been conflicting. In SGA neonates, some observational studies did not show any correlation between cord blood total cholesterol concentration and birth weight, while others reported either positive or negative statistically significant correlations between these parameters [54, 55]. Similarly, study findings regarding total cholesterol concentration of prepubertal SGA children have been conflicting [56, 57]. Thus, in adolescents born SGA, a metanalysis showed a weak but statistically significant inverse association between total cholesterol and birth weight [58]. In another metanalysis which included pubertal children born SGA as well as adults born SGA, impaired fetal growth was shown to correlate negatively with total cholesterol concentration [59].

In the present metanalysis, neonates born SGA demonstrated lower concentration of HDL cholesterol compared to those born AGA. However, in prepubertal and pubertal children HDL cholesterol concentration did not differ significantly between SGA and AGA individuals. Inconsistent evidence regarding HDL cholesterol concentration in SGA neonates stems from observational studies [54, 60]. While a large multi-cohort study in pubertal children and adults has reported lower HDL concentration in SGA compared to their AGA peers [56], a metanalysis found no association between birth weight and HDL cholesterol concentration [59].

Furthermore, in this metanalysis, no statistically significant difference in LDL cholesterol concentration was found between neonates, prepubertal and pubertal SGA children and their respective AGA peers. Similarly, previous metanalyses, including studies in pubertal children and adults, have found no association between birthweight and circulating LDL cholesterol concentration [58, 59, 61].

In addition, in the present metanalysis, neonates born SGA showed greater concentration of triglycerides, compared to neonates born AGA, while there was no difference among prepubertal and pubertal children born SGA compared to their AGA peers. This finding is in accordance with previous observational studies which reported increased triglyceride concentration in SGA neonates compared to AGA controls [54, 60]. Notably, certain retrospective studies reported elevated triglyceride concentration in prepubertal children born with low birthweight [56, 57]. In addition, review studies have reported a possible negative correlation between birthweight and triglyceride concentration in prepubertal children and adolescents [61].

This study is the first metanalysis comparing neonates, prepubertal and pubertal children born SGA to neonates, prepubertal and pubertal children born AGA, regarding their carbohydrate and lipid metabolism. The methodology of the study appropriately follows the PRISMA statement and is registered in PROSPERO. This metanalysis has several limitations that should be considered when interpreting results. Although study groups were stratified by age, some variability may still exist within these groups due to different developmental stages of the included children, which could influence the metabolic outcomes. Obviously, differences in laboratory techniques and measurement protocols may affect the comparisons of the results across studies as well. To address this heterogeneity, a random-effects model was applied in the statistical analysis. For certain outcomes, the number of eligible studies was limited, reducing thus, the statistical power and rendering impossible a meta-regression analysis. Despite these limitations, the findings of this metanalysis provide valuable clinical insights into the metabolic consequences associated with the SGA status and highlight the need for further research in this field. In conclusion, regarding carbohydrate metabolism, prepubertal children born SGA present greater concentration of insulin, compared to prepubertal children born AGA, while regarding lipid metabolism, neonates born SGA, present lower concentration of HDL and greater concentration of triglycerides compared to neonates born AGA. Children born SGA are at increased risk for altered carbohydrate and glucose metabolism due to epigenetic changes occurring *in utero*, which eventually accompany them in postnatal life and might be at the origin of ailments in adult life.

## Supplementary Information

Below is the link to the electronic supplementary material.

Supplementary Material 1

## Data Availability

No datasets were generated or analysed during the current study.

## Abbreviations

BMI: Body Mass Index
CG: Catch-up Growth
CVD: Cardiovascular Disease
GA: Gestational Age
IUGR: Intrauterine Growth Restriction
IVGTT: iv Glucose Tolerance Test
LBW: Low Birth Weight
MTT: Milk Tolerance Test
NBW: Normal Birth Weight
NCG: No Catch-up Growth
N/A: Not Applicable
OGTT: Oral Glucose Tolerance Test
