# Supplementary material for: Carbohydrate and lipid metabolism in neonates and children born SGA. A systematic review and metanalysis
Source: Endocrine. 2025 Sep 1;90(3):1120–37. doi: 10.1007/s12020-025-04402-9 (PMC12708704; doi:10.1007/s12020-025-04402-9)
Supplement: Supplementary file 1 — Supplementary Material 1 [file 12020_2025_4402_MOESM1_ESM.docx]

**Carbohydrate and Lipid Metabolism in Neonates and Children born SGA. A Systematic review and Metanalysis**

**Journal name:** Endocrine

Kalliopi Kissoudi^1^, Christos Chatzakis^2^, Panagiotis Christos Mastorakos^3^, Maria Papagianni^4^, Alexandros Sotiriadis^2^, George Mastorakos^5, *^

^1^ First Department of Obstetrics and Gynecology, Aristotle University of Thessaloniki, 54642 Thessaloniki, Greece

^2^ Second Department of Obstetrics and Gynecology, Aristotle University of Thessaloniki, 54642 Thessaloniki, Greece

^3^ Leeds University, Leeds, United Kingdom

^4^ 3rd Department of Pediatrics, Ippokratio Hospital of Thessaloniki, Aristotle University of Thessaloniki, 54642 Thessaloniki, Greece.

^5^ Unit of Endocrinology, Diabetes Mellitus and Metabolism, Aretaieion Hospital, Athens Medical School, National and Kapodistrian University of Athens, 11528 Athens, Greece.

* Corresponding author: George Mastorakos, [mastorakg@gmail.com](mailto:mastorakg@gmail.com)

**Table S1**

Excluded studies with reason for exclusion

| **Study; Year** | **Reason for exclusion** |
| --- | --- |
| Sidiropoulou et al; 2018 | Gender-based outcomes |
| Bavdekar et al; 1999 | Multiple BW groups |
| Abe et al; 2007 | No comparison population |
| Boyne et al; 2010 | No comparison population |
| Cai et al; 2007 | No comparison population |
| de Kort et al; 2010 | No comparison population |
| Faienza et al; 2013 | No comparison population |
| Léniz et al; 2021 | No comparison population |
| Lupinska et al; 2023 | No comparison population |
| Polo Perucchin et al; 2011 | No comparison population |
| Sayers et al; 2009 | No comparison population |
| Stawerska et al; 2013 | No comparison population |
| Stevens et al; 2014 | No comparison population |
| Szałapska et al; 2010 | No comparison population |
| de Leeuw et al; 1976 | No full text available |
| van der Steen et al; 2016 | No full text available |
| Yada et al; 2003 | No full text available |
| Yang et al; 2024 | No full text available |
| de la Calzada et al; 2009 | Review article |
| Desai et al; 1995 | Review article |
| Geremia et al; 2006 | Review article |
| Hernández et al; 2011 | Review article |
| Jaquet et al; 2003 | Review article |
| Maiorana et al; 2007 | Review article |
| Ozanne et al; 1999 | Review article |
| Tappy et al; 2006 | Review article |
| Yajnik et al; 2004 | Review article |
| Byberg et al; 2000 | Wrong outcome |
| Dufour et al; 2011 | Wrong outcome |
| Hirschler et al; 2008 | Wrong outcome |
| Iniguez et al; 2006 | Wrong outcome |
| Jornayvaz et al; 2004 | Wrong outcome |
| Malpique et al; 2019 | Wrong outcome |
| Tenhola et al; 2005 | Wrong outcome |
| Alonso-Larruscain et al; 2019 | Wrong population |
| Arends et al; 2005 | Wrong population |
| Barg et al; 2013 | Wrong population |
| Boscaini et al; 2015 | Wrong population |
| Brøns et al; 2012 | Wrong population |
| Cho et al; 2014 | Wrong population |
| Domínguez Hernández et al;2016 | Wrong population |
| Eyzaguirre et al; 2012 | Wrong population |
| Hofman et al; 1997 | Wrong population |
| Hokken-Koelega et al; 2003 | Wrong population |
| Ibanez et al; 2011 | Wrong population |
| Kim et al; 2006 | Wrong population |
| Krishnaveni et al; 2014 | Wrong population |
| Lemos et al; 2010 | Wrong population |
| Maguolo et al; 2021 | Wrong population |
| Mi et al; 2000 | Wrong population |
| Perälä et al; 2011 | Wrong population |
| Phillips et al; 1998 | Wrong population |
| Poulsen et al; 1997 | Wrong population |
| Pulzer et al; 2001 | Wrong population |
| Reinehr et al; 2009 | Wrong population |
| Reinehr et al; 2010 | Wrong population |
| Spencer et al; 1997 | Wrong population |
| Stroescu et al; 2013 | Wrong population |
| van der Kaay; 2009 | Wrong population |
| Verkauskiene et al; 2008 | Wrong population |
| Vestbo et al; 1996 | Wrong population |
| Viera et al; 2021 | Wrong population |
| Willemsem et al; 2008 | Wrong population |
| Yu et al; 2021 | Wrong population |

Reasons for exclusion: (n=65): Full texts not available (n = 4); review article (n = 9); wrong population (n = 30); wrong outcome (n = 7); no comparison population (n =12); multiple BW groups (n =2); gender-based outcome (n = 1)
